# Do Musicians and Non-musicians Differ in Speech-on-Speech Processing?

**DOI:** 10.3389/fpsyg.2021.623787

**Published:** 2021-02-18

**Authors:** Elif Canseza Kaplan, Anita E. Wagner, Paolo Toffanin, Deniz Başkent

**Affiliations:** ^1^Department of Otorhinolaryngology/Head and Neck Surgery, University Medical Center Groningen, University of Groningen, Groningen, Netherlands; ^2^Research School of Behavioral and Cognitive Neurosciences, Graduate School of Medical Sciences, University of Groningen, Groningen, Netherlands

**Keywords:** speech-in-noise, musical training, visual world paradigm, pupillometry, eye-tracking, speech-on-speech

## Abstract

Earlier studies have shown that musically trained individuals may have a benefit in adverse listening situations when compared to non-musicians, especially in speech-on-speech perception. However, the literature provides mostly conflicting results. In the current study, by employing different measures of spoken language processing, we aimed to test whether we could capture potential differences between musicians and non-musicians in speech-on-speech processing. We used an offline measure of speech perception (sentence recall task), which reveals a post-task response, and online measures of real time spoken language processing: gaze-tracking and pupillometry. We used stimuli of comparable complexity across both paradigms and tested the same groups of participants. In the sentence recall task, musicians recalled more words correctly than non-musicians. In the eye-tracking experiment, both groups showed reduced fixations to the target and competitor words’ images as the level of speech maskers increased. The time course of gaze fixations to the competitor did not differ between groups in the speech-in-quiet condition, while the time course dynamics did differ between groups as the two-talker masker was added to the target signal. As the level of two-talker masker increased, musicians showed reduced lexical competition as indicated by the gaze fixations to the competitor. The pupil dilation data showed differences mainly in one target-to-masker ratio. This does not allow to draw conclusions regarding potential differences in the use of cognitive resources between groups. Overall, the eye-tracking measure enabled us to observe that musicians may be using a different strategy than non-musicians to attain spoken word recognition as the noise level increased. However, further investigation with more fine-grained alignment between the processes captured by online and offline measures is necessary to establish whether musicians differ due to better cognitive control or sound processing.

## Introduction

Musical training may grant normal-hearing listeners an advantage in auditory tasks, not only for tasks related to music, but also in encoding and processing speech in adverse listening conditions (e.g., Kraus and Chandrasekaran, 2010), such as in the presence of competing talkers (e.g., Başkent and Gaudrain, 2016). However, various studies that investigated whether long-term musical training improves speech-in-noise processing yielded conflicting results (for a review, see Coffey et al., 2017). Inconsistency in findings is fueled not only by the challenges to reproducibility across paradigms and labs but also by different levels of complexity in signal-noise properties (Swaminathan et al., 2015), as well as considerable individual variability that is inherent to speech processing in adverse listening conditions (e.g., Peelle, 2018). In the current study, we used three measures, one that gives a post-task measure (offline), and two online measures that capture real-time processing of speech perception (gaze-fixations) and cognitive resources employed (pupillometry). We used stimuli of comparable complexity across different measures of spoken language processing within the same group of participants. With this approach, we aimed to investigate whether we can find differences in speech-on-speech processing between musicians and non-musicians consistent within groups across all measures.

Music and language both engage complex cognitive processes that result from the interaction of attention, memory, motor, and auditory systems. Musical training requires extensive use of domain-specific, as well as domain-general auditory processes (Asaridou and McQueen, 2013), and has been investigated as a potential learning process that increases cross-domain plasticity (Pantev and Herholz, 2011). According to Patel (2014), musical training may improve cross-domain plasticity and strengthen the shared neural resources required for processing both music and speech, and this may enhance auditory attention and auditory working memory (Besson et al., 2011). For normal hearing listeners with typical language development, speech processing without background noise is considered an automatic process (Shiffrin and Schneider, 1977) that does not pose extra demands on cognitive resources (Marslen-Wilson and Tyler, 1981). Nonetheless, in adverse listening conditions, speech processing may become demanding (Mattys et al., 2012; McQueen and Huettig, 2012; Heald and Nusbaum, 2014). Accordingly, musical training has been suggested as a means to enrich speech-in-noise perception, through the recruitment of these strengthened cross-domain resources and mechanisms when processing becomes challenging (Strait and Kraus, 2011).

In the past decades, there has been an increase in the number of studies that investigate speech-on-speech processing in musicians and non-musicians (Parbery-Clark et al., 2009; Ruggles et al., 2014; Boebinger et al., 2015; Swaminathan et al., 2015; Başkent and Gaudrain, 2016; Clayton et al., 2016; Madsen et al., 2017, 2019; Yates et al., 2019; Bidelman and Yoo, 2020). However, this line of research has provided ambiguous results. Parbery-Clark et al. (2009) showed that musicians performed better than non-musicians in the often used audiological measures of Hearing in Noise Test (HINT; Nilsson et al., 1994), which measures sentence recognition in speech-shaped noise at a variety of adaptive signal-to-noise ratios, and QuickSIN (Killion et al., 2004), which measures sentence recall in four-talker babble presented at fixed target-to-masker ratios (TMRs). On the contrary, Ruggles et al. (2014) failed to replicate their results with a larger sample size. Particularly for speech-on-speech perception, Başkent and Gaudrain (2016) showed in a sentence recall task that musicians correctly recall more words than non-musicians in a single-talker masker. Similarly, Deroche et al. (2017) observed musicians to have better speech reception thresholds in a two-talker masker, but not in non-speech maskers. On the other hand, Madsen et al. (2017, 2019) did not find any group differences in a variety of speech-in-noise and speech-on-speech conditions with a larger sample size.

The nature of the demands imposed by both the task and the stimuli may play a role in the variability across different results reported in the literature. Sentence recall without any background noise involves encoding and retention of auditory information. Without a secondary task, the recall performance is automatic and effortless, with no involvement of central executive functions (Baddeley et al., 2009). In the presence of a secondary task, attention plays a role in retention and retrieval of information (Treisman, 1964). Inhibiting the interference from the speech from competing talkers or processing the acoustic information in the target signal may serve as such a secondary task, and thus speech-on-speech requires additional central executive involvement. The interference from the masker also depends on the type of the background masker itself, i.e., lexical content of the speech masker (Helfer and Jesse, 2015); amount of linguistic interference from the speech masker (Calandruccio et al., 2010); number of talkers in speech masker, such as 1, 2, and 4 vs. 16 talkers (Rosen et al., 2013), and this may lead to variations in the chunking strategies to inhibit the background talkers (Miller, 1947; Bronkhorst, 2015; Calandruccio et al., 2017). Thus, regardless of the musical experience, the type of target and masker properties used across different studies and the nature of the task demands (i.e., recall vs. recognition) might play a role in the “different findings” in the literature.

In contrast to most studies that employed offline behavioral measures, studies that sought musician/non-musician differences using online measures have more consistently found a difference between groups. Offline measures, such as accuracy of responses, are obtained after the task and do not capture individual’s real time processing of the stimuli. Online measures provide real-time information while the spoken language processing happens (Godfroid, 2019). Most online measures that have been employed in testing differences between musicians and non-musicians in speech-in-noise related tasks used neuroimaging methods, such as EEG (Parbery-Clark et al., 2009; Meha-Bettison et al., 2018) or MEG (Puschmann et al., 2018). These methods require precise control over stimuli, such as phonemes embedded within broadband noise (Parbery-Clark et al., 2012; Du and Zatorre, 2017) or within multi-talker babble noise (Strait and Kraus, 2011), and hence measure lower level sound encoding. In addition, many of these studies used different methods (e.g., ABR, EEG, and MEG) and even different exact dependent variables within a single method (e.g., latencies vs. phase-locking vs. peak magnitude in ABR).

Eye-tracking is another online method that captures the real-time, automatic, and anticipatory information processing (Allopenna et al., 1998). Cooper (1974) has shown that when listeners are presented simultaneously with spoken language and a screen that depicts objects that are mentioned in the utterance, listeners perform a visual search on the screen and fixate their gaze upon the objects mentioned in the utterance. Eye movements and gaze fixations thus reveal the incremental processing of spoken language as the speech signal unfolds over time. In addition to capturing gaze fixations, it is also possible to record pupil responses with an eye-tracker. Pupil dilation is taken to reflect changes in the engagement of cognitive resources next to the quick ocular reflexes to changes in luminance (Beatty, 1982). Changes in pupil dilation have been used as a measure of attention and effort (Kahneman, 1973) and have been applied also to record mental effort in language processing (Kuchinsky et al., 2013; Schmidtke, 2014; Wagner et al., 2016; Nagels et al., 2020). Whereas pupil responses due to changes in luminance take about 150–400 ms (Bergamin et al., 2003), pupil responses that relate to cognitive processing are slower and can take about 1 s (Hoeks and Levelt, 1993; Wierda et al., 2012). An increase in pupil dilation is often considered to reflect increased cognitive effort and increased allocation of attentional resources.

In the current study, we used two online measures: (1) gaze-tracking, which provides insight into spoken word recognition in real time and (2) pupillometry, which provides insight into the employment of cognitive resources in spoken word recognition in the presence of speech maskers. We also implemented a sentence recall task that gives an offline measure, utilizing similar sets of stimuli in both online and offline measures, and the same groups of non-musician and musician participants. The purpose was to test whether the results from the online and offline measures would all reveal processing differences between groups.

The first experiment is a sentence recall task, in which participants listen to, recall, and repeat target Dutch sentences presented with two-talker Dutch sentence maskers in different TMRs. This offline task provides an estimate of intelligibility by measuring the percentage of correctly recalled words, similar to the study reported by Başkent and Gaudrain (2016). These authors showed that musicians overall had a larger number of correctly identified words than non-musicians when the target sentence was embedded in a single-talker masker. We aimed to further test whether the difference these authors observed would be present also when using a similar task with slightly different sets of stimuli and more effective masking conditions, with parameters adjusted to not reach ceiling performance across different TMRs. According to Rosen et al. (2013) masking effects differ when the number of background talkers changes from 1 to 2 or 4, and Calandruccio et al. (2017) showed that a two-talker masker was the most effective masker. The similarity between the masker and target also plays a role in how strong the masking effect can be. The more dissimilar the two streams are in terms of the target and masker speakers (Brungart et al., 2001), target and masker speakers’ voices (Darwin et al., 2003), the language of the target and the masker (Lecumberri and Cooke, 2006), and the semantic content of the target and the masker (Calandruccio et al., 2010), the easier it becomes to understand the target speech stream. Thus, in the current study, based on literature and confirmed by an initial pilot study for sufficient masking effects, we have decided to use two-talker maskers of same sex talkers as background noise, and a talker of same sex for the target speaker. We hypothesized that if musical training benefits speech-on-speech perception, as some of the literature has suggested, musicians would recall more words correctly when compared to non-musicians in the two-talker masked sentence recall task.

**Figure 1:**
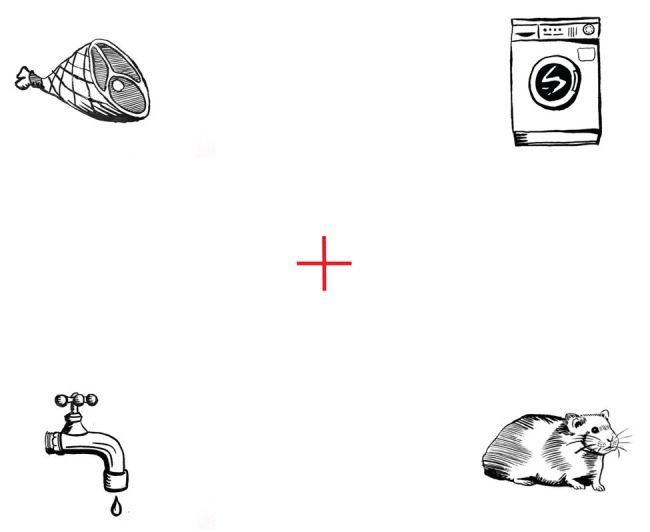
Example of the screen presented during the experiment, where ham (**upper left**) is the competitor word and hamster (**lower right**) is the target word. The illustrations were made by Jop Luberti for the purpose of this study. This image is published under the CC BY NC 4.0 license.

The second experiment is an eye-tracking experiment that employed visual-world paradigm (VWP; Eberhard et al., 1995; Tanenhaus et al., 1995; Allopenna et al., 1998; Salverda and Tanenhaus, 2017), where we measured participants’ gaze-fixations and pupil dilation (Wagner et al., 2016; Nagels et al., 2020). In the VWP, while listening to target sentences embedded in two-talker masker sentences, participants visually search for and choose the image of a target word uttered by the target speaker. Spoken word recognition involves ambiguity resolution among lexically related items (see also a TRACE model: McClelland and Elman, 1986; Salverda et al., 2003). As listeners hear the acoustic speech cues coming from the target speaker, they form and continually fine-tune hypotheses regarding the target word. The displayed images include the target word and a phonological competitor that shares an onset-overlapping segment with the target word (Figure 1) and two unrelated distractors. The linking hypothesis is that the shifts in visual attention among the objects displayed on the screen are a consequence of what is heard in the utterance and can capture real-time spoken language processing (Cooper, 1974; Allopenna et al., 1998; Salverda and Tanenhaus, 2017). Hence, the time course of gaze fixations to the images of the target and competitor words can capture the time course of the continual integration of acoustic information, while the signal is mapped to meaning. Additionally, changes in pupil dilation reflect how the cognitive resources allocated for spoken language processing are affected by the presence of the two-talker masker.

In the second experiment, we aimed to answer three main questions: (1) to what extent would speech maskers affect processes underlying spoken word recognition, such as lexical decision making, as captured by the time course of gaze fixations, (2) how would the effect of masking on spoken word recognition differ between musicians and non-musicians, and (3) do musicians and non-musicians allocate their cognitive resources differently when processing speech in two-talker masker vs. speech-in-quiet, as captured by the changes in pupil dilation. For the first question, we hypothesized that there would be an effect of masking on the time course of gaze fixations, in line with previous literature that utilized a similar paradigm to test the effect of signal degradation on lexical decision making (McQueen and Huettig, 2012; Wagner et al., 2016). For the second question, we assumed that musicians might be better able to focus their attention on the target or suppress the background noise. If that is the case, we hypothesized that the differences observed in the time course of gaze fixations to the competitor to differ less between speech-in-quiet and speech in two talker masker conditions for musicians than for non-musicians. For the third question, the effect of masking on the employment of cognitive resources that can be captured by pupil dilation, we expected to find differences between musicians and non-musicians in the two-talker masker condition but not in speech-in-quiet, as this would be the control condition, where the spoken language processing is assumed to occur automatically and in a similar manner in both groups.

## Experiment 1: Sentence Recall Experiment

In this experiment, participants repeated a grammatically correct and meaningful Dutch target sentence embedded within two-talker maskers that also consisted of meaningful Dutch sentences. Different utterances of the same target and masker speakers were utilized in both this experiment and the eye-tracking experiment in order for the stimuli to be consistent between the online and the offline measures. Participants completed the eye-tracking experiment (Experiment 2) first to become familiar with the voice of the target speaker.

### Method

#### Participants

Sixteen musicians (10 females) and 17 non-musicians (11 females) from Groningen, the Netherlands, participated in the study and met the inclusion criteria. All participants were native Dutch speakers that were raised monolingually (i.e., did not have a second language spoken by their caregivers at home). The musicians were selected based on the following criteria (Parbery-Clark et al., 2009; Fuller et al., 2014; Başkent and Gaudrain, 2016): having started musical training at/before the age of seven, having at least 10 years of musical training, and actively practicing music for at least 3 years prior to the study (the years of musical training do not refer to active years of engagement, but rather indicate the amount of formal training). Accordingly, the non-musician criteria were the following: not meeting all of the musician criteria, and additionally, not having more than 3 years of musical training (see Table 1 below for musical background information of participants).

**Table 1:** Musical training background (in years).

	Years of training	Age onset year	Current age
Musicians			
1	25	7	45
2	23	4	32
3	15	6	29
4	14	5	23
5	14	7	30
6	13	6	26
7	12	6	22
8	12	6	21
9	11	7	22
10	11	6	33
11	10	6	29
12	10	7	20
13	10	7	22
14	10	5	33
15	10	7	19
16	10	7	28
Mean	13.13	6.19	27.13
Non-musicians			
1	3	8	21
2	3	6	22
3	3	8	27
4	3	10	20
5	2	8	25
6	1.5	8	19
7	1	10	34
8	1	16	20
9	1	56	57
10	none	none	23
11	none	none	22
12	none	none	21
13	none	none	24
14	none	none	25
15	none	none	21
16	none	none	46
17	none	none	21
Mean	2.05	14.4	26.35

To ensure that participants had normal hearing, we assessed audiometric thresholds to make sure the hearing levels were <20 dB HL for pure tone thresholds measured at audiometric frequencies from 250 to 4,000 Hz for both ears. We used modified criteria that differed than the standard clinical audiometric measurements that include 8,000 Hz, since some musicians had unilaterally higher thresholds than 20 dB HL due to playing an instrument close to the ear (i.e., violin). All participants reported having normal or corrected-to-normal vision, i.e., using contact lenses or glasses, and having no language disorders. The study was approved by the Medical Ethics Committee of the University Medical Center Groningen. All participants were given information about the study prior to participation, they provided written consent, and after that, their hearing was screened and music and language background inclusion criteria were tested before experiments started. At the end of the study, they were given financial compensation for participation according to department guidelines.

#### Apparatus

Participants were seated at a 50 cm distance in front of a 17-inch computer screen in a sound attenuated booth. Both experiments were set up and ran in MATLAB (The MathWorks), using the PsychToolBox (Kleiner et al., 2007). The auditory stimuli were presented through an AudioFire4 sound card (Echo Digital Audio Corporation) and played on a Tannoy Precision 8D speaker (Tannoy Ltd) located behind the computer screen, in front of which the participants were seated. For the sentence recall task, participants followed instructions through a computer screen and used a keyboard to proceed within the experiment. Their verbal responses were recorded through another laptop using Audacity (version 2.1.3.0).

#### Materials and Design

Twenty-eight semantically neutral Dutch target sentences were embedded in two-talker maskers. We used the filler items recorded for Wagner et al. (2016) as the target sentences. Target sentences were uttered by a female Dutch speaker without any regional accent (f0 mean = 173.91 Hz, sd = 44.03 Hz). Each target sentence contained seven to nine words.

The masker sentence set consisted of meaningful Dutch sentences from the corpus of Versfeld et al. (2000), uttered by a different female speaker than the target speaker (f0 mean: 160.01 Hz, sd: 54.83 Hz). This female speaker’s utterances were used to generate two-talker maskers. All target and masker sentences were root-mean-square normalized in intensity.

The target sentences were embedded within the two-talker maskers, where the target sentence onset was 500 ms after the masker onset and the target offset was 500 ms before the masker offset, similar to Başkent and Gaudrain (2016). The 200 ms at the beginning and at the end of the maskers were ramped up and down, respectively, to avoid audible clicks. If the duration of a single masker sentence was not sufficient to cover the duration of target duration plus 1,000 ms, another sentence was added to the masker sequence to add up to the required total duration.

Four lists of sentences were generated corresponding to the four levels of TMRs. The TMR levels (−3, −5, −7, and −9 dB) were chosen based on Calandruccio et al. (2017) and our own pilot study. Each TMR condition contained seven sentences, with either 54 or 55 words in total. The lists were generated so that each sentence was presented in all TMR conditions across participants. The two-talker masker was fixed at 75 dB SPL presentation level, while the single-talker target’s presentation level was adjusted depending on the TMR condition.

#### Procedure

The experiment started with a practice phase, where the participants completed four trials corresponding to the four TMR levels included in the experimental phase. The participants completed the eye-tracking experiment first, to become familiar with the voice of the target and masker speakers before moving on to the sentence recall task. To help the participant with identifying which speech stream from the target masker combination was the target speech, it was explained that the target voice was the same as the female speaker from the eye-tracking experiment and that the target speaker began to speak later than the masker speaker. During both practice and experimental phases, the participants were instructed to verbally repeat the utterance of the target speaker immediately at the end of each trial. The participants’ verbal responses were recorded to be coded later for the correctly recalled words. The experimental phase contained 28 sentences in total, all presented in presence of two-talker masker (seven sentences per list × four TMR levels). The TMR conditions were presented in a random order across participants to prevent a potential effect of the order of presentation.

#### Data Analysis

Two independent Dutch-native speaker student assistants assessed the correctness of the recalled words from the recordings of the participant responses. The student assistants were blind to the hypotheses and rationale of the experiment but not to the group belonging of the participants since the group assignment made part of how participants were coded. Every word within the sentence was used to calculate the correct scores. Morphological changes (i.e., in tense and plural marker) were taken into account when giving a correct score. The response was considered correct if minor mistakes were made, such as using unstressed use of a pronoun (zij – ze [she]), different forms of modals (can-could) or diminutive forms of nouns (addition of -je). Percentage of correctly recalled words per each trial per participant was calculated by dividing the correctly recalled words by the total amount of words contained in the sentence.

R (R Core Team, 2013) and lme4 (Bates et al., 2015) were used to perform a generalized linear mixed effects analysis. The optimal model was determined in an iterative backward fitting with model comparison of *χ*^2^ test and evaluation of Akaike’s information criterion (Akaike, 1974; Baayen, 2008). The most complex model including all fixed effects with interactions and maximal random effects structure (Barr et al., 2013) was constructed. Then, the interaction term is removed to check for the effect of the interaction. If the interaction was not significant, the main effect of the fixed effects in the model was evaluated by removing each fixed effect from the full model. We followed Barr et al. (2013) in simplifying the random effects structure until the model converged. The best model was determined by model comparison and evaluated by likelihood ratio test through ANOVA Chi-Square tests.

### Results

**Figure 2:**
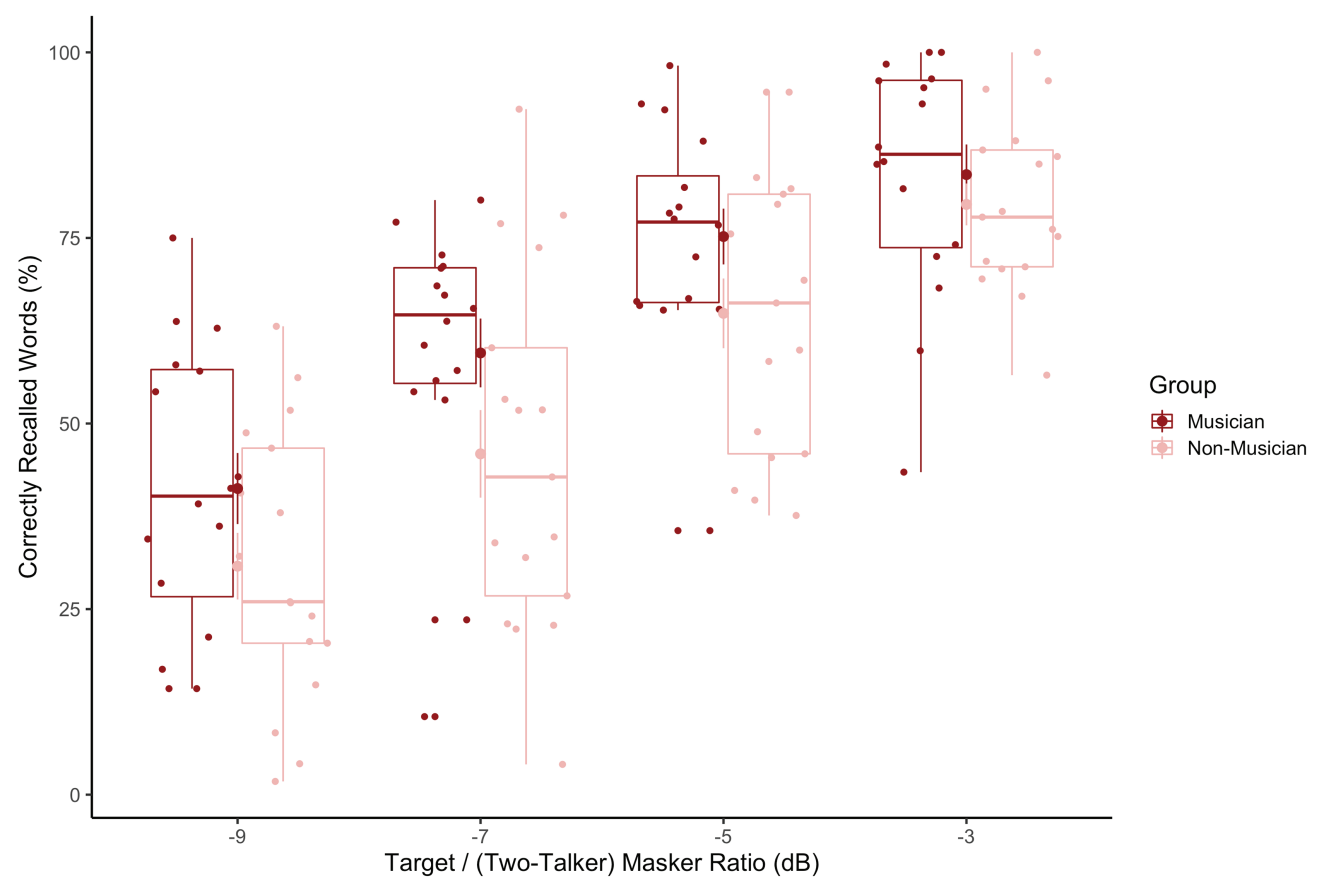
Boxplots represent the percentage scores of correctly recalled words for musicians (dark red) and non-musicians (light pink) across Target-to-Masker Ratios (from −9 dB **most left** to −3 dB **most right**). The horizontal lines in the boxes represent the median value and the dots represent data points for individual participants.

Figure 2 illustrates the percentage of correctly recalled words averaged across participants and across different TMR conditions. The percentage of correctly recalled words was calculated separately for each participant, target sentence, and TMR condition, and was used as the dependent measure in the generalized linear mixed effect model. TMR Conditions (−3, −5, −7, and −9 dB) and Group (musician vs. non-musician) were entered as fixed effects. The final model that converged resulted in having a model with two random intercepts: per subject and per sentence. Step-wise model comparison revealed that the interaction term between TMR Conditions and Group did not improve the model significantly [*χ*^2^(3) = 2.58, *p* = 0.46, AIC difference = 3]. The main effects of both TMR Conditions [*χ*^2^(3) = 181, *p* < 0.000, AIC difference = −175] and Group were significant [*χ*^2^(1) = 4.3, *p* = 0.038, AIC difference = −2]. Table 2 shows the converted predicted probabilities of the fixed effect model estimates using plogis function in R (R Core Team, 2013; also see Supplementary Materials for the full model summary). Table 3 shows the 95% confidence intervals which were determined *via* bootstrap resampling based on 1,000 simulations. The bootstrapped confidence intervals do not cross zero indicating that TMR Condition and Group are significant predictors in our model. Overall, the two groups’ performances differed with musicians recalling more words correctly across TMR conditions. Also, as the TMR value became lower, both groups’ recall performance became worse. The lack of interaction between group and TMR condition was not significant, thus not supporting the claim that musicians’ performance would improve more than non-musicians’ performance as the task became more difficult.

**Table 2:** The percentage of correct recall as predicted by the final model across target-to-masker-ratio (TMR) conditions for both groups.

Plogis transformed model estimates for the % of correctly recalled words by both groups		
TMR condition (dB)	Musicians (%)	Non-musicians (%)
−3	92.4	85.8
−5	82.7	70.3
−7	63.7	46.5
−9	35.6	21.5

**Table 3:** Bootstrapped estimates for the 95% confidence intervals, based on resampling of the sentence recall model.

Bootstrap resampling: confidence intervals		
	2.50%	97.50%
.sig01	0.48	1.08
.sig02	0.67	1.34
(Intercept)^*^	1.83	3.32
TMR = −5 dB	−1.52	−0.43
TMR = −7 dB	−2.50	−1.41
TMR = −9 dB	−3.71	−2.56
Group: non-musician	−1.41	−0.09

* Intercept: group = musician, TMR = −3 dB.

## Experiment 2: Eye-Tracking Experiment

In the eye-tracking experiment (online measure), implementing the visual world paradigm (Cooper, 1974; Salverda and Tanenhaus, 2017), participants identified a target word, uttered in a sentence by the target speaker, among four images displayed on the screen (Figure 1). This paradigm enables to measure the time course of lexical decision making by capturing the gaze fixations to the target and competitor images. The target speaker’s utterance was either presented without background talkers (speech-in-quiet) or was embedded within two-talker masker. Gaze fixations enabled us to capture how the process of lexical decision making would be affected by two-talker maskers. In addition, we also recorded participants’ pupil dilation, which is taken to reflect how the cognitive effort and changes in allocation of cognitive resources imposed by the two-talker maskers would differ between groups.

### Method

#### Participants

The same participants as in the sentence recall task also completed the visual world paradigm.

#### Apparatus

The experiment was conducted in the same sound attenuated booth as in the sentence recall task with the same setup, and with the additional use of an eye-tracker. Eye-Link II head-mounted eye-tracker (SR-research), with a sampling rate of 250 Hz, was used for collecting gaze and pupil responses. The presentation of the stimuli was controlled with MATLAB (The MathWorks) and the PsychToolBox (Kleiner et al., 2007). The gaze fixations and pupil responses were recorded utilizing the Eyelink Toolbox for MATLAB (Cornelissen et al., 2002). The eye-tracker had two cameras that were placed in front of the participants’ eyes. Since saccades are synchronized, recordings were collected from only one eye as it is sufficient to capture the gaze fixations and as monocular recordings are more accurate than binocular recordings (Godfroid, 2019). The eye-tracker camera was placed in front of the participants’ right eye to record the gaze movements and pupil dilations. The lighting in the room was kept constant throughout the experiment.

#### Materials and Design

Utterances of the same target and masker speakers from the sentence recall task were used. The sentence list contained different sentences than the sentence recall task to avoid stimulus repetition, i.e., the participants were not familiar with the utterances but they were with the voices of the target and masker speakers. The target speakers’ sentence set was taken from the same database as the sentence recall task that was also previously used in Wagner et al. (2016). Target sentences consisted of 36 grammatical, semantically neutral Dutch sentences. Each target sentence contained a *target word* that was either monosyllabic or polysyllabic, which shared an onset-overlapping syllable with a *competitor word* (Table 4).

**Table 4:** Example of sentences containing both a polysyllabic and a monosyllabic target word.

Sentence	Target	Competitor
Ik zag een *beitel* op de grond liggen.	beitel	bij
*(I saw a chisel lying on the floor.)*	(chisel)	(bee)
Hij zei dat die *kom* gevallen was.	kom	computer
*(He said that the bowl had fallen.)*	(bowl)	(computer)

Each sentence was played through a loudspeaker behind the screen while four pictures were displayed on the screen simultaneously. The images utilized in the visual world paradigm were taken from the same data set that was used and validated previously in Wagner et al. (2016). The images were displayed in four regions of interests at the four corners of the screen defined by dividing the screen into three vertical and three horizontal lines, with the middle sections being narrower than the others. A fixation cross appeared in the middle of the screen in each trial. The images consisted of black-and-white static drawings, and the background of the screen was always kept white, so that the color of the screen did not change, to prevent potential pupil responses that may be caused by the changes in lumination. In each trial the image referring to the target word, its’ phonological competitor and two distractors that were semantically and phonologically unrelated to the target and the competitor word was displayed simultaneously as the auditory stimuli was presented. For example, if the target word was hamster, the phonological competitor would be ham (Figure 1).

Target sentences were presented either in quiet (without any background talker) or were embedded within two-talker maskers at either 0 or −5 dB TMR. These TMRs were within the range of TMRs tested in the sentence recall task, but differed slightly in the exact values, as they were newly chosen based on a pilot study in order to ensure that participants achieved above 75% accuracy in all conditions of the visual world paradigm. This was necessary to elicit sufficient number of correct trials that could be included in the analysis of gaze fixation data. We used the same procedure as in the sentence recall task to generate target sentences embedded within two-talker maskers.

The experiment had two blocks: speech-in-quiet and speech in two-talker masker. The speech-in-quiet block was presented first, and the two-masker conditions (0 and −5 dB TMR) were presented in random order within one block. In order to counterbalance the presentation of all sentences across conditions and participants, we generated six lists. Each list contained 12 sentences and was assigned to one of the three conditions (speech-in-quiet, 0 dB TMR, −5 dB TMR).

#### Procedure

Prior to starting the experiment, and similar to procedures of Wagner et al. (2016), participants were shown the pictures utilized in the visual world paradigm and asked to name them, in order to make sure they would correctly identify the images during the experiment. If they named a picture differently, they were told by the experimenter how it would be referred to in the experiment. After the familiarization was completed, participants were asked to move to the booth. Before data collection, the eye-tracker was calibrated for each participant. Throughout the experiment, there was a drift check every five trials and if necessary the eye-tracker was recalibrated.

The experiment consisted of a practice phase and two experimental phases: speech-in-quiet and speech in two-talker masker. In the practice phase, participants completed four trials each in the quiet and masked conditions. In the experimental phase, participants heard 12 sentences in speech-in-quiet block and 24 sentences in speech in two-talker masker. The sentences within a block were presented in a random order for each participant. Each trial began with a red cross appearing in the middle of the screen. It was followed by the simultaneous presentation of both the auditory and visual stimuli. Participants were instructed to pay attention to the voice of the speaker they heard in the speech-in-quiet condition, which was the target speaker, throughout the experiment. They used the mouse to choose the image of the target word from the four pictures displayed on the screen. Participants were also instructed to blink as little as possible during the trial. Between each trial, they were given written instruction to blink and were asked to press space bar when ready to start the next trial. They were not given feedback on their response accuracy. Following the practice phase, the experimental phase always started with the speech-in-quiet block for the participants to become familiar with the voice of the target speaker. Upon completing the speech-in-quiet condition, participants could take a break and the eye-tracker was recalibrated. For the speech in two-talker masker condition, the two TMR levels were presented in random order within a block.

#### Data Analysis

##### Pre-Processing

As was aimed for by design and by a pilot study, all participants at all conditions scored above 75% accuracy in target word picture detection. Similar to Wagner et al. (2016), trials with inaccurate target detection (in total 5.3% of all trials – musicians 4.90%, non-musicians 5.67%, and no significant difference between groups) and trials that contained blinks longer than 300 ms (1.00% of all accurate trials) were excluded from the data analysis. Blinks shorter than 300 ms were linearly interpolated from the median value of the 25 samples preceding the blink to the median value of the 25 samples following the blink. Following the interpolation procedure, the data were binned into intervals of 20 ms by averaging five consecutive samples.

##### Gaze Fixations

Four regions of interest were defined that corresponded to where the target, competitor, and two distractor images appeared, by dividing the screen in *x*-*y* coordinates. A fifth region existed between the four regions and along the line of the fixation cross. The gaze fixations were recorded in these *x*-*y* coordinates along with the pupil size for every sample. At any given time the fixation was coded as 1 at the region it was observed and as 0 at the remaining regions. These responses were then used to calculate the proportions of gaze fixations to the images displayed and to generate the time course of gaze fixations as the speech information unfolded in time. Since it takes about 150–200 ms for a saccade to be planned and executed upon receiving the auditory information (Hallett, 1986), in our analysis of gaze fixations, we used the interval starting from 200 to 1,800 ms after the onset of the target word was included in the data analysis. The offset end of the interval was taken as 1,800 ms, since the gaze fixations to the image referring to the target are stable and the process of lexical decision making is captured by that point.

The fixations to all images signify how the spoken language processing occurs and how it changes due to increased uncertainty when the two-talker masker is added to the signal. In the present experiment, however, we operationalized changes in lexical competition across conditions through changes in gaze fixations to the competitor image along the time course of lexical decision making. Therefore, the time course of gaze fixations to the competitor was modeled as time series data in growth curve models (Mirman, 2014). The gaze fixations to the competitor reflected information regarding the timing of lexical decision making, and the effect of the two-talker masker on this process within both groups. This multilevel regression enables modeling of change in the proportion of fixations across time by using orthogonal polynomials. R (R Core Team, 2013) and lme4 (Bates et al., 2015) were used to model the time curves within the interval 200–1,800 ms after the onset of the target word. The model selection procedure was identical to that of the sentence recall task. The time course curves for target and competitors were compared across conditions (speech-in-quiet, 0 dB TMR, and −5 dB TMR) and groups (musician vs. non-musician).

##### Pupil Dilation

In line with Wagner et al. (2019), we quantified changes in pupil dilation by computing event-related pupil dilation (ERPD) and we also quantified the changes relative to the resting state pupil dilation according to the formula below:

$$\%ERPD=\frac{observation-baseline}{baseline}*100$$

In the ERPD formula above, processes attributed to the resolution of lexical ambiguity between the target and the competitor can be computed by replacing “observation” with all the pupil dilation data recorded between 0 and 3,000 ms after the onset of the target word. A longer time window is selected than the gaze fixations, as the pupil changes due to cognitive processes take about 1 s to occur (Hoeks and Levelt, 1993; Wierda et al., 2012). “Baseline” is replaced with the average pupil dilation measured pre-target, between −200 and 0 ms before the onset of the target word. Each percentage of change in the ERPD was calculated for each trial and participant.

In addition, we calculated the resting state normalized pre-target baselines to control for how the pre-target baselines changed in relation to the initial state of the participants before each block. The resting state baseline consisted of the average of a 4 s of pupil data recorded before each experimental block began. The same ERPD formula as above was used to calculate relative change in the pre-target baselines in relation to the resting baseline. Observation was replaced with the pre-target baselines and the baseline was replaced with resting state baseline.

We modeled the time course changes of ERPD as time series data in growth curve models (Mirman, 2014). The time window that was used to model the time curves was chosen between 0 and 3,000 ms after the onset of the target word. The window of analysis starts earlier than for the gaze fixations for it to be aligned with the end point of the baseline and the baseline is taken as a point where the listener has not yet heard the target word. This interval is different than that of the gaze-fixations time interval, since pupil response reflecting spoken language processing takes longer than the gaze fixation. The model selection procedure was identical to that described for the sentence recall task.

### Results

#### Gaze Fixations

**Figure 3:**
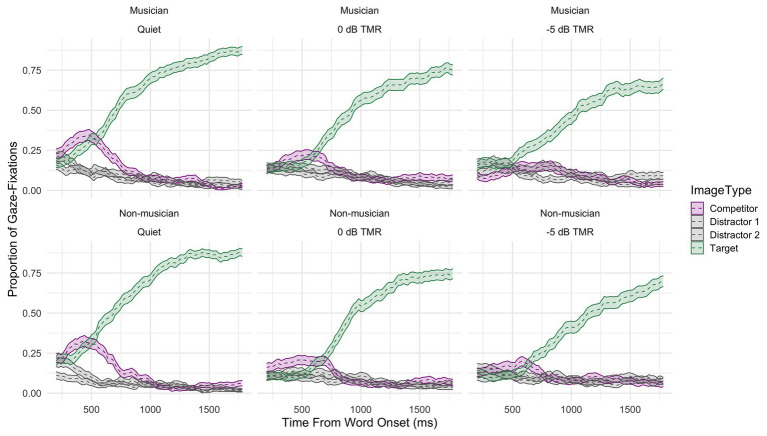
The raw data of time course curves of gaze fixations averaged across participants and items to both the target (green), competitor (purple), and distractors (gray) shown for all conditions (from left to right: speech-in-quiet, 0 dB TMR, and −5 dB TMR) and both groups (top panels: musicians, and bottom panels: non-musicians).

Figure 3 shows the proportions of gaze fixations averaged across participants to the targets and competitors across conditions: speech-in-quiet (left panel), and TMR = 0 dB (middle panel), TMR = −5 dB (right panel), and groups: musicians (top panels), and non-musicians (bottom panels). The upper lines in green represent the proportion of fixations to the target word, the lower lines in purple represent the proportion of fixations to the competitor, and the gray lines represent the proportion of fixations to the distractor images, each shown with 95% confidence intervals. The overall certainty in decision making reflected by the proportion of gaze fixations to the target decreased gradually for both groups as the masker was added and the level of masking increased.

The competitor time course curves were modeled in a logistic regression with probability of fixations to the phonological competitor as fourth order polynomials with the following terms: linear term (the overall slope of the curve), quadratic term (symmetricity in the rise and fall around a central inflection point, i.e., the peak in curve), cubic term, and quartic term (steepness of the curvature around inflection points; Mirman, 2014). The model intercept referred to the overall average fixation proportion. The dependent variable in the model was the proportion of fixation to the competitor. The fixed effects in the model included Condition (Speech-in-Quiet, 0 dB TMR, and −5 dB TMR), Group (musician vs. non-musician), and the four polynomial terms used to define the time-course curves. Upon conducting iterative backward model selection, the final model included the four terms of the polynomial, as well as interactions between Condition and Group and a three-way interaction between Condition and Group and each term of the polynomial function (See Supplementary Material for a summary of model estimates). The maximal random-effects structure that converged for the present analysis included by-subject and by-item random intercept and random slopes for all polynomial terms. The three-way interaction between Condition, Group, and the four terms that describe the time course curves indicates that the probability of gaze fixations to the competitor changed between conditions differently between groups of musicians and non-musicians on all the terms that describe the polynomial. The model fit was significantly improved by adding the interaction between Condition and Group and the linear term [*χ*^2^(2) = 34.48, *p* < 0.001, AIC difference = −30.48], quadratic term [*χ*^2^(2) = 21, *p* < 0.001, AIC difference = −17.01], cubic term [*χ*^2^(2) = 26.26, *p* < 0.001, AIC difference = −22.26], and quartic term [*χ*^2^(2) = 13.80, *p* = 0.001, AIC difference = −9.81].

**Figure 4:**
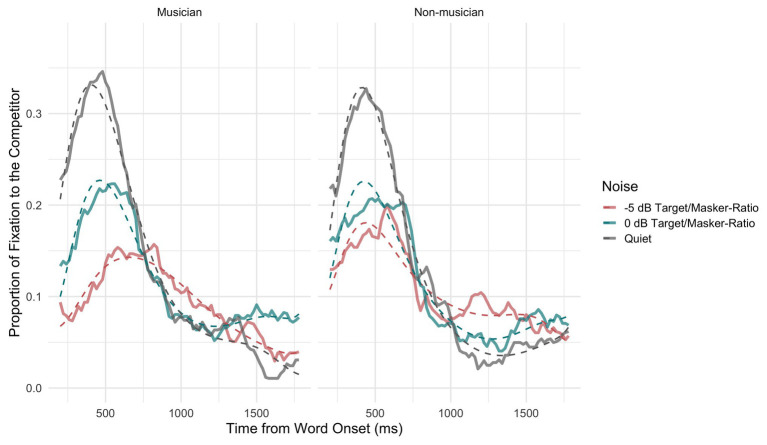
Probability of gaze fixations to the competitor averaged across participants and items shown for musicians (**left panel**) and non-musicians (**right panel**) for the three conditions (speech-in-quiet: gray, 0 dB TMR: turquoise, and −5 dB TMR: red) as observed in the data (solid lines) and predicted by the model (dashed lines).

Figure 4 shows both the averaged proportion of fixations to the competitor (solid lines) and the probability of fixations to the competitor (dashed lines) from the fitted model for both musicians (left) and non-musicians (right) and the different conditions (speech-in-quiet: gray, 0 dB TMR: turquoise, and −5 dB TMR: orange). In the speech-in-quiet condition, the two groups did not differ in the model intercept (*β* = 0.01, SE = 0.24, *z* = 0.03, *p* = 0.97). This was in line with our prediction that both groups would exhibit similar fixations to the competitor in speech-in-quiet. The overall average fixation proportions differed for the two groups as the intensity of the two-talker maskers increased. Figure 4 shows that the curve’s peak was more widely spread and lower in height in −5 dB TMR for musicians than for non-musicians. This difference is supported by the significance of the three way interaction in the cubic (*β* = −3.03, SE = 0.61, *z* = −4.95, *p* < 0.001) and quartic terms (*β* = −2.00, SE = 0.60, *z* = −3.57, *p* < 0.001). This could indicate that musicians exhibited less lexical competition and in turn resolved the ambiguity faster by fixating on the target image earlier (see Figure 3) when the two-talker masker became higher in intensity in −5 dB TMR. The aggregated time course curves for non-musicians were not as smooth and did not fit the model as well as the data for musicians did in this condition. This suggests that the non-musician group was behaving less homogeneously than the musicians. This difference in −5 dB TMR condition could also indicate that the two groups use different strategies when processing speech in two-talker masker, which could have affected the incremental process of lexical competition.

#### Pupil Dilation

Percentage of change in event related pupil dilation was first calculated per participant, trial and condition with pre-target baseline in the ERPD formula. The time series of ERPDs was analyzed by means of growth curve analysis, same as that of the gaze fixations method, by fitting third order orthogonal polynomials. The dependent variable in the model was the ERPD calculated based on pre-target baseline (−200–0 ms preceding the onset of the target word). The fixed effects in the final model included Condition (speech-in-quiet, 0 dB TMR, and −5 dB TMR), Group (musician vs. non-musician), and the three orthogonal terms used to describe the curves. The maximal random-effects structure that converged for the present analysis included by-subject and by-item random intercept and random slopes for all polynomial terms. Upon conducting iterative backward model selection, the final model included the three terms of the polynomial, as well as interactions between Condition and Group and a three-way interaction between Condition and Group and each term of the polynomial function (See Supplementary Material for a summary of final model estimates). The three-way interaction between Condition, Group and the terms that describe the time course curve showed that musicians’ and non-musicians’ task ERPDs were affected differently across conditions. The model fit was significantly improved by adding the interaction between Condition and Group and the linear term [*χ*^2^(2) = 23.91, *p* < 0.001, AIC difference = −19.91], quadratic term [*χ*^2^(2) = 36.01, *p* < 0.001, AIC difference = −32.01], and cubic term [*χ*^2^(2) = 16.05, *p* < 0.001, AIC difference = −12.05].

**Figure 5:**
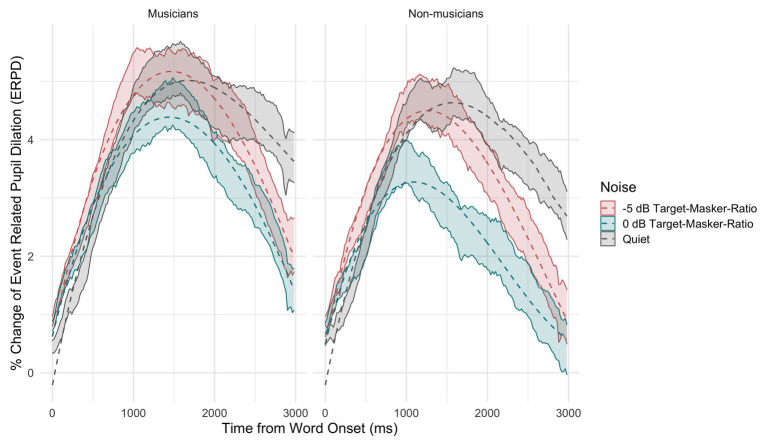
Percentage of change in the observed pre-target baseline normalized event-related pupil dilation (ERPDs; transparent ribbon line with 95% CI) and as predicted by the model (solid lines), averaged across participants and items, shown for musicians (**left panel**) and non-musicians (**right panel**) across conditions (speech-in-quiet: gray, 0 dB TMR: turquoise, and −5 dB TMR: red).

Figure 5 shows the percentage of change in ERPDs (solid lines with transparent filling) and the fitted model output (dashed lines) across time, for musicians (left) and non-musicians (right) across three conditions (speech-in-quiet: gray, 0 dB TMR: turquoise, and −5 dB TMR: orange). The three-way interaction suggests that both groups’ task-related pupil changes that are assumed to reflect the lexical decision making differed between speech-in-quiet versus speech in two-talker masker conditions. Overall, the model intercept that reflects the area under the curve was highest for the speech-in-quiet condition and did not differ significantly between groups (*β* = −0.43, SE = 0.74, *p* = 0.56). There was a gradual decrease in the overall area under the curve from speech-in-quiet to −5 dB TMR and lastly to 0 dB TMR for both groups. The greatest difference in the area under the curve between the two groups was in the 0 dB TMR (*β* = −0.66, SE = 0.05, *p* < 0.001). The change in the ERPD in 0 dB TMR for non-musicians occurred faster initially, reaching the peak earlier and releasing from the increase in pupil dilation slower over time when compared to musicians, as indicated by the three-way interaction of 0 dB TMR with quadratic (*β* = 3.44, SE = 0.64, *p* < 0.001) and cubic terms (*β* = 2.55, SE = 0.64, *p* < 0.001).

We have also looked at the pre-target baseline changes in relation to the resting baseline between groups and conditions to further investigate the effect of processing speech in two-talker masker. To compare the percentage of change relative to the resting state baseline, we firstly compared whether the resting state baselines at the beginning of each experimental block differed between groups. The mean resting baseline consisting of the average pupil size recorded in pixels at the beginning of each experimental block began was 3001.53 for musicians (*sd* = 316.41), 2908.74 for non-musicians (*sd* = 389.38) preceding the speech-in-quiet block and 2902.89 for musicians (*sd* = 266.33), 2781.51 for non-musicians (*sd* = 364.27) preceding the speech in two talker masker block. We performed an equivalence test using Bayesian t-test from the BayesFactor package in R (Rouder et al., 2009). The Bayes factor of the resting baseline comparison between groups before speech-in-quiet block was 0.41 and the before the speech in two talker masker block was 0.53. According to Jeffreys (1961), Bayes factors between 0 and 1.10 are “not worth more than a bare mention.” Therefore, the resting state pupil baselines are taken not to differ between groups at the beginning of each experimental block.

**Figure 6:**
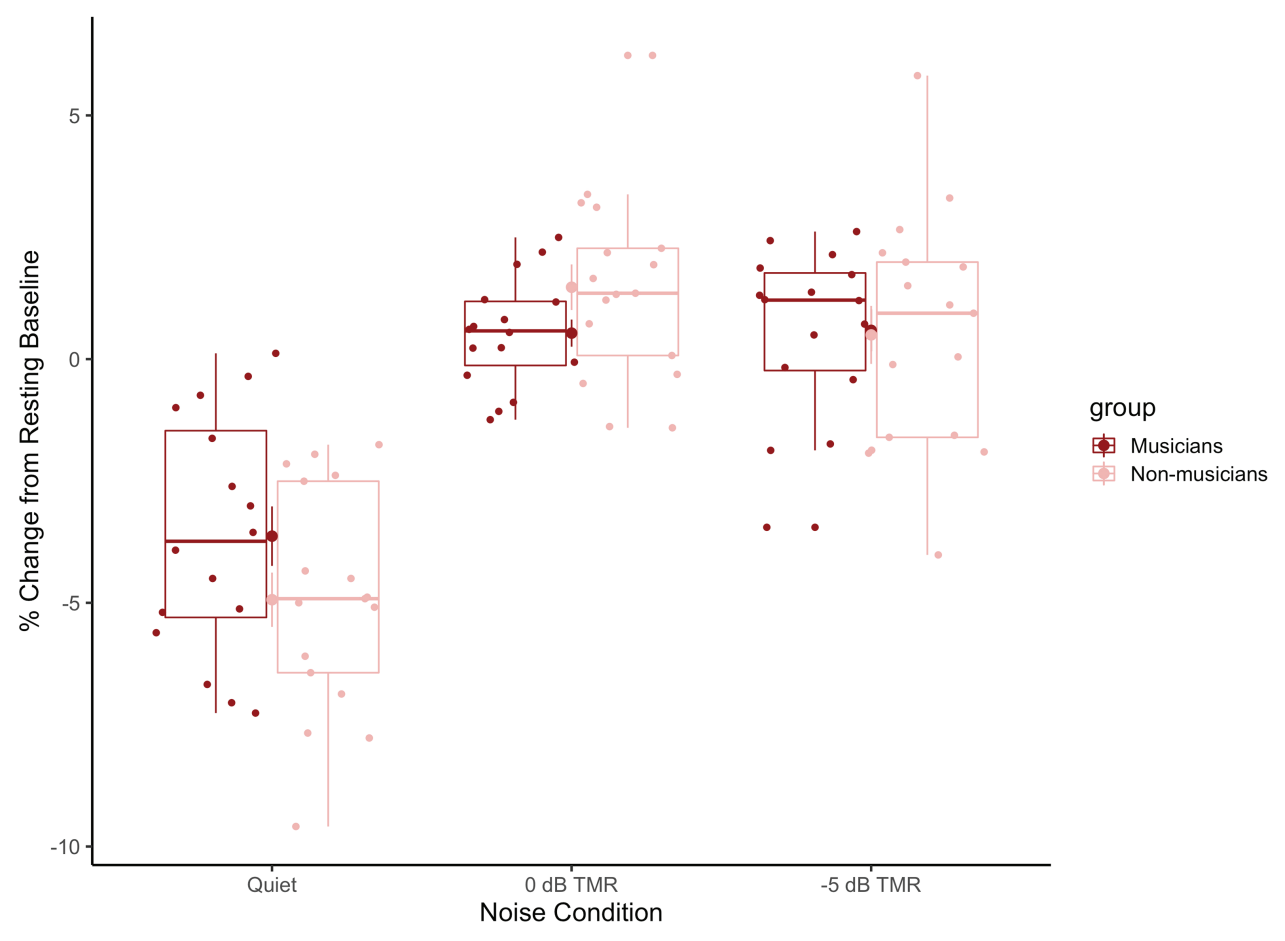
Boxplots represent the percentage of change in the pre-target baseline relative to the resting state baseline for musicians (dark red) and non-musicians (light pink) across conditions (speech-in-quiet: left, 0 dB TMR: middle, and −5 dB TMR: right). The horizontal line in the box represents the median values and the dots represent the individual participants’ relative change in pre-target baseline averaged across trials.

Figure 6 shows the mean percentage of change in the pre-target baselines in relation to resting state baselines for musicians (dark red) and non-musicians (light pink) across conditions (speech-in-quiet: left, 0 dB TMR: middle, and −5 dB TMR: right). This figure shows that in the speech-in-quiet block, the pupil dilation in the pre-target baseline reduced over the course of that experimental block (the average change in this block is below 0 for both groups). We do not see this pattern for the speech in two-talker masker block, where the average change relative to the resting state pupil size remains above 0 for both groups. In line with Wagner et al. (2019), we interpret this as a result of processing speech in the presence of two-talker masker. The smaller decrease in pupil size within that block suggests overall increase in sustained attention as participants were processing masked speech.

We ran a linear mixed model with the interaction of the Condition (quiet, 0 dB TMR, −5 dB TMR) and Group (musician vs. non-musicians) with the percentage change in the resting baseline relative pre-target baselines as the dependent measure. The random structure included a by-subject intercept and random slope of condition by subject. The backward model comparison revealed an interaction between Condition and Group [*χ*^2^(2) = 1767.3, *p* < 0.001, AIC difference = −1,763]. The significant two-way interaction between 0 dB TMR and group indicates that the two groups mainly differed in 0 dB TMR condition in terms of the relative change in pre-target baseline (*β* = 2.25, SE = 0.86, *t* = 2.60, *p* = 0.014).

Figure 6 shows the mean percentage of change in the pre-target baselines in relation to resting baselines for musicians (dark red) and non-musicians (light pink) across conditions (speech-in-quiet: left, 0 dB TMR: middle, and −5 dB TMR: right). We ran a linear mixed model with the interaction of the Condition (quiet, 0 dB TMR, and −5 dB TMR) and Group (musician vs. non-musicians) with the percentage change in the resting baseline relative pre-target baselines as the dependent measure. The random structure included a by-subject intercept and random slope of condition by subject. The backward model comparison reveled an interaction between Condition and Group [*χ*^2^(2) = 1767.3, *p* < 0.001, AIC difference = −1,763]. The significant two-way interaction between 0 dB TMR and group indicates that the two groups mainly differed in 0 dB TMR condition in terms of the relative change in pre-target baseline (*β* = 2.25, SE = 0.86, *t* = 2.60, *p* = 0.014).

## General Discussion

The current study examined whether and in what ways speech-on-speech perception differed between musicians and non-musicians. Previous literature showed that there may be an effect of musical experience on speech perception in the presence of speech maskers, yet, the findings have not been consistent across studies that used offline measures (reflecting the post-task performance after speech processing is completed; e.g., Başkent and Gaudrain, 2016; Madsen et al., 2017). Here, we aimed to test whether both the offline and the online measures would reveal differences in speech-on-speech processing between musicians and non-musicians. We used speech materials taken from the same set of recordings for both experiments, and participants took part in both experiments in one session. Results from both experiments showed that there is a difference between the musician and non-musician groups’ responses when processing speech in two-talker masker. In the sentence recall task, musicians overall performed better than non-musicians, having recalled more words correctly, similar to Başkent and Gaudrain (2016). Moreover, the lack of a significant interaction between group and TMR condition did not support Strait and Kraus (2011) findings that musicians’ performance may be better than non-musicians’ performance as the task became more demanding. In contrast, in the eye-tracking experiment, the main difference in terms of the time course of gaze fixations was in the masking condition of −5 dB TMR, i.e., when the masker level was higher; at this TMR, musicians exhibited fewer looks to the lexical competitor and resolved the lexical ambiguity faster than non-musicians. This suggested that the musicians may be employing a different strategy than non-musicians when processing speech in the presence of speech maskers. The pupil dilation related to the task-relevant processes (lexical decision making) occurred in a similar manner between the two groups, whereas the effect of two-talker masker mainly was observed in 0 dB TMR condition.

In the sentence recall task, musicians overall performed better, having recalled more words correctly than non-musicians. In line with previous studies, our results also showed within-group variation (e.g., Swaminathan et al., 2015; Başkent and Gaudrain, 2016). In speech-in-noise perception, as the similarity between the target/noise properties increases, it becomes more difficult to segregate the target/noise streams (see Table 1 in Schubert and Schultz, 1962). This in turn may lead to more variable ways to disentangle the target and noise streams, especially when the background noise becomes more similar to the speech signal, as in speech-on-speech perception (Lutfi et al., 2003; Kidd and Colburn, 2017). Hence, speech-on-speech studies tend to yield more variable results compared to speech-in-noise tasks in general (Lutfi et al., 2003; Festen and Plomp, 2004). We also observed inter-individual variability in our results, but this variation was more pronounced in the non-musician group. The selection criteria may have played a role; while musicians are selected based on strict criteria, non-musicians domain-general auditory skills are not controlled for. In future studies, it could be useful to approach musicality as a spectrum or a continuous variable (e.g., Puschmann et al., 2018) or to check for standardized musicality indexes (e.g., Goldsmith Musical Sophistication Index, Müllensiefen et al., 2014). Since the participants of the present study had been initially selected based on the criteria of the previous literature (Parbery-Clark et al., 2009; Fuller et al., 2014), we could not perform such analysis during or after the study.

Online measures of spoken language processing obtained with an eye-tracker enabled us to capture language processing in real-time. The results from the VWP showed that the lexical decision making process did not differ between the two groups in the speech-in-quiet condition, in terms of the timing of the amount of lexical competition and timing of ambiguity resolution. When the two-talker masker was added to the target speech signal, both groups had decreased amounts of fixations to the competitor, but musicians particularly showed this effect in the −5 dB TMR condition. In the −5 dB TMR condition, musicians looked less at the competitor and the visual inspection of the time course curve for the target image also indicated that musicians started looking at the target word’s image earlier in this condition. We interpret this as musicians making the lexical decision faster in −5 dB TMR condition when compared to both non-musicians and their own speech-in-quiet condition results. According to the models of spoken language recognition, lexical decision making occurs in an incremental manner. Based on the speech signal, multiple lexical hypotheses are activated based on the weights that the acoustic input refers to (e.g., cohort competitors vs. rhyme competitors). As the signal is degraded or masked due to background noise, the activation is spread across different candidates and none of them may reach the threshold to trigger activation sufficient to elicit lexical competition (e.g., Wagner et al., 2016). Both groups’ exhibited decreased fixations to the competitor word, as well as the target, as the two-talker maskers level increased. This could also be caused by the participants looking at the fixation cross more and waiting until enough information is accumulated, which is referred to as “wait-and-see” strategy by McMurray et al. (2017), while trying to understand the target speaker in two-talker masker. Musicians appear to disambiguate the target signal earlier; however, it appears that the incremental process of lexical competition that took place in speech-in-quiet for both groups is reduced (does not seem to be taking place) in −5 dB TMR for musicians. This could indicate that musicians might be using a different strategy to accommodate to the background noise and attain the task goal. For example, they may be employing a different strategy to increased uncertainty with altered criteria for activation of the nodes in the process of lexical decision making (McQueen and Huettig, 2012). It would be clearer to test this hypothesis with more controlled manipulations of the acoustic cues in the target speech stream to infer whether musical training enables attending to the target stream, suppressing of the background noise or simultaneous processing of multiple streams of auditory information.

We measured listeners’ change in pupil dilation, in order to gain more insights into how the process of lexical decision making that occurs effortlessly in speech-in-quiet is affected by the addition of two-talker masker to the signal, as well as how the employment of cognitive resources differs between groups. We looked at the change in pupil dilation that captured task-relevant processes attributed to the resolution of lexical ambiguity and lexical decision making between the target and the competitor. The results revealed that this process occurred in a similar way for both groups across conditions. This is reasonable since only the accurate responses were taken into consideration, where the participants were performing the goal of the task as was intended. The main difference between groups was in 0 dB TMR condition. Non-musicians exhibited less dilation when compared to musicians. The speech in two-talker masker conditions did not generate larger changes in the time course of pupil dilation when compared to speech-in-quiet, as the pre-target baselines already had more dilated pupils as the speech processing before the target word occurred in two-talker masker. For both groups, the speech in two-talker masker elicited similar pupil dilation in the speech-in quiet condition. In the masked condition, the processing of speech signal required additional resources (1) for extracting the target signal from background talkers or inhibiting the background talkers and (2) to resolve the lexical competition (Wagner et al., 2016; Peelle, 2018; Nagels et al., 2020). This difference in processing can also be seen in the relative changes in the pre-target baselines (Figure 6). The pre-target baselines were overall higher when processing speech in two-talker masker when compared to the speech-in-quiet by both musicians and non-musicians. This indicates that processing effort had already increased before the onset of the target word for both groups in the speech in two-talker masker conditions. The largest observed difference between the two groups’ the pre-target baselines was in 0 dB TMR, with the non-musicians having higher pre-target baselines. This could have been the cause of the lower pupil response observed in 0 dB TMR (Figure 5). Since both 0 and −5 dB TMRs were presented within one block, it is not possible to disentangle the source of the difference peculiar to 0 dB TMR. Taken together, the higher pre-target baselines for speech in two-talker masker conditions indicate that speech maskers increased the cognitive effort for both groups, whereas, the difference was larger for non-musicians in the 0 dB TMR easier noise level.

Overall, the combination of gaze fixations and pupil responses suggest that musicians are employing a different strategy than non-musicians when processing masked speech in the way they resolve lexical ambiguity, especially in the lower TMR level that presents a more difficult task. This is not in line with the sentence recall findings, where the increase in masker intensity did not reveal a larger difference in performance between musicians and non-musicians; across all TMR levels tested, they performed better than non-musicians and to a seemingly similar level. These results can have several explanations: there may be differences between musicians and non-musicians in how they encode or process information in the speech streams, they may differ in how they suppress the background masking talkers, or the two groups may differ in how quickly and efficiently they implement a strategy to deal with the added noise. Another possibility to explain the observed difference between groups may be that musicians encode and process both streams of information simultaneously, as suggested in a recent MEG study (Puschmann et al., 2018). Puschmann et al. (2018) have reported that in a selective listening task, where a continuous target speech stream is presented along with a competing speaker that needs to be disregarded, the number of years of musical training was strongly correlated with the ability to track the masker stream and make use of both streams to achieve the task goals. In addition, the study by Puschmann et al. (2018) revealed that musicians’ neural responses also attuned to the distractor stream as well as the target speaker. Our results indicate that there is a difference between musicians and non-musicians when processing speech-on-speech. However, it requires further empirical work, with more targeted manipulations of acoustic properties of the stimuli, in order to infer whether the observed group difference is due to better ability to uptake the acoustic information in the target stream or to suppress the background noise, or alternatively to process both streams in parallel.

## Conclusion

In the present study, we investigated whether and in what ways musicians differed from non-musicians in speech-on-speech perception and processing. Both our offline and online measures of speech perception indicated that musicians and non-musicians differed in processing speech presented within a two-talker masker. Musicians overall performed better in the sentence recall task. It should be noted that our results do not imply a causal link between musical training and speech-on-speech perception. Such a claim would require a specially designed musical training program and a longer period of testing. In addition, further empirical work with different samples of participants and different sets of target/masker properties are required to clarify whether these results are generalizable to different listening situations.

Gaze-tracking results revealed that both groups did not differ in the speech-in-quiet condition. Once background noise was added in terms of two-talker masker, musicians and non-musicians differed in how the time course of gaze fixations was affected by masking, especially as the noise level increased. The pupil dilation had increased for both groups in the speech in two-talker masked condition when compared to speech-in-quiet. Overall, all results combined, we have observed differences between musicians and non-musicians in both performances of speech perception in two-talker speech masker, and in the time course of lexical decision making in noise. Although the online measure of eye-tracking enabled us to capture that musicians may be using a different strategy as the noise level increased, it requires further empirical work to determine whether the effects observed were due to musicians having better cognitive control regarding processing of both the target and the masker streams or due to a difference in the sound encoding and retrieval in general.

## Data Availability Statement

The datasets presented in this study can be found in online repositories. The names of the repository/repositories and accession number(s) can be found at: https://doi.org/10.34894/YFEHFJ.

## Ethics Statement

The studies involving human participants were reviewed and approved by The Medical Ethical Committee (METc) of the UMCG. The patients/participants provided their written informed consent to participate in this study.

## Author Contributions

EK, AW, and DB conceptualized and designed the study. EK collected the data. EK, PT, and AW conducted data analysis and interpretation. PT assisted with the programming of the experiment. EK wrote the original draft. All authors critically assessed and approved the final manuscript.

### Conflict of Interest

The authors declare that the research was conducted in the absence of any commercial or financial relationships that could be construed as a potential conflict of interest.
