# Supplementary material for: Do Musicians and Non-musicians Differ in Speech-on-Speech Processing?
Source: Front Psychol. 2021 Feb 18;12:623787. doi: 10.3389/fpsyg.2021.623787 (PMC7931613; doi:10.3389/fpsyg.2021.623787)
Supplement: Supplementary file 1 [file Data_Sheet_1.pdf]

Supplementary Materials

Final Model Estimates

Sentence Recall Model

```
## Generalized linear mixed model fit by maximum likelihood (Laplace
##   Approximation) [glmerMod]
##  Family: binomial  ( logit )
## Formula: CorrectPerc ~ condition + group + (1 | ID) + (1 | item)
##
##
## Fixed effects:
##              Estimate Std. Error z value Pr(>|z|)
## (Intercept)   2.4975     0.3664   6.817 9.29e-12 ***
## -5 dB TMR    -0.9315     0.2619  -3.557 0.000375 ***
## -7 dB TMR    -1.9360     0.2618  -7.395 1.42e-13 ***
## -9 dB TMR    -3.0913     0.2809 -11.005  < 2e-16 ***
## non-mus      -0.7028     0.3286  -2.139 0.032472 *
## ---
## Signif. codes:  0 '***' 0.001 '**' 0.01 '*' 0.05 '.' 0.1 ' ' 1
##
## Correlation of Fixed Effects:
##            (Intr) cndtn5 cndtn7 cndtn9
## -5 dB TMR  -0.440
## -7 dB TMR  -0.481  0.605
## -9 dB TMR  -0.495  0.581  0.640
## non-mus    -0.494  0.016  0.043  0.062
```

The model intercept: *Group: musicians, Condition -3 dB TMR*

Competitor Model

```
## Generalized linear mixed model fit by maximum likelihood (Laplace
##   Approximation) [glmerMod]
##  Family: binomial  ( logit )
## Formula: competitor ~ (linear + quadratic + cubic + quartic) * condition *
##          group + (linear + quadratic + cubic + quartic | ID) +
##          (linear + quadratic + cubic + quartic | item)
##
##
## Fixed effects:
##                          Estimate Std. Error z value Pr(>|z|)
## (Intercept)             -2.730901   0.194201 -14.062  < 2e-16 ***
## linear                 -11.586070   1.237986  -9.359  < 2e-16 ***
## quadratic               -0.726812   1.021950  -0.711 0.476960
## cubic                    0.806694   0.751877   1.073 0.283313
## quartic                 -2.263242   0.566093  -3.998 6.39e-05 ***
## 0TMR                     0.146021   0.045580   3.204 0.001357 **
## 5TMR                    -0.097440   0.048015  -2.029 0.042421 *
## non-mus                  0.007226   0.240618   0.030 0.976042
## linear:0TMR              6.301480   0.441840  14.262  < 2e-16 ***
## linear:5TMR              6.322817   0.483385  13.080  < 2e-16 ***
## quadratic:0TMR           1.603940   0.452459   3.545 0.000393 ***
## quadratic:5TMR          -2.134695   0.482183  -4.427 9.55e-06 ***
## cubic:0TMR               1.268148   0.432681   2.931 0.003380 **
## cubic:5TMR               0.647130   0.461090   1.403 0.160473
## quartic:0TMR             0.452016   0.382931   1.180 0.237837
## quartic:5TMR             2.229415   0.413687   5.389 7.08e-08 ***
## linear:non-mus           1.974719   1.373244   1.438 0.150435
## quadratic:non-mus        2.637945   1.130213   2.334 0.019594 *
## cubic:non-mus            2.467928   0.785660   3.141 0.001682 **
## quartic:non-mus          0.595722   0.694407   0.858 0.390955
## 0TMR:non-mus            -0.191482   0.063694  -3.006 0.002645 **
## 5TMR:non-mus             0.142505   0.065812   2.165 0.030361 *
## linear:0TMR:non-mus     -3.346302   0.585020  -5.720 1.07e-08 ***
## linear:5TMR:non-mus     -1.456366   0.629050  -2.315 0.020603 *
## quadratic:0TMR:non-mus  -2.297392   0.592854  -3.875 0.000107 ***
## quadratic:5TMR:non-mus  -0.093761   0.627564  -0.149 0.881234
## cubic:0TMR:non-mus      -2.146264   0.579526  -3.703 0.000213 ***
## cubic:5TMR:non-mus      -3.025624   0.610736  -4.954 7.27e-07 ***
## quartic:0TMR:non-mus    -0.459037   0.527514  -0.870 0.384197
## quartic:5TMR:non-mus    -1.995721   0.559758  -3.565 0.000363 ***
## ---
## Signif. codes:  0 '***' 0.001 '**' 0.01 '*' 0.05 '.' 0.1 ' ' 1
```

***The model intercept:** Group: musicians, Condition: Speech-in-Quiet*

ERPD Pre-Target Baseline Normalized Model

```
## Linear mixed model fit by maximum likelihood  ['lmerMod']
## Formula: erpd ~ (linear + quadratic + cubic) * condition * group +
## (linear + quadratic + cubic | ID) + (linear + quadratic + cubic | item)
##
##
## Fixed effects:
##                          Estimate Std. Error t value
## (Intercept)               3.91342    0.58013   6.746
## linear                   10.20026    2.95478   3.452
## quadratic               -12.43285    2.14526  -5.795
## cubic                     2.30824    1.19375   1.934
## 0TMR                     -0.70869    0.03639 -19.474
## 5TMR                     -0.10222    0.03711  -2.754
## non-mus                  -0.43148    0.74201  -0.581
## linear:0TMR              -9.39725    0.45038 -20.865
## linear:5TMR              -8.11141    0.45912 -17.667
## quadratic:0TMR           -0.07603    0.45339  -0.168
## quadratic:5TMR           -2.15097    0.46211  -4.655
## cubic:0TMR               -1.16410    0.45579  -2.554
## cubic:5TMR               -0.75512    0.46437  -1.626
## linear:non-mus           -3.03707    3.79816  -0.800
## quadratic:non-mus        -0.13149    2.87103  -0.046
## cubic:non-mus            -0.14977    1.60082  -0.094
## 0TMR:non-mus             -0.66377    0.05104 -13.004
## 5TMR:non-mus             -0.14598    0.05211  -2.801
## linear:0TMR:non-mus      -3.07846    0.63162  -4.874
## linear:5TMR:non-mus      -1.75780    0.64468  -2.727
## quadratic:0TMR:non-mus    3.43980    0.63574   5.411
## quadratic:5TMR:non-mus   0.24811    0.64877   0.382
## cubic:0TMR:non-mus       2.55403    0.63865   3.999
## cubic:5TMR:non-mus       1.40929    0.65163   2.163
```

***The model intercept:** *Group: musicians, Condition: Speech-in-Quiet*

Resting Baseline Normalized Pre-Target Baseline Model

```
## Linear mixed model fit by REML. t-tests use Satterthwaite's method [
## lmerModLmerTest]
## Formula: restbasenorm ~ condition * group + (1 + condition | ID)
##    Data: dat
##
## REML criterion at convergence: 984711.6
##
## Scaled residuals:
##     Min      1Q  Median      3Q     Max
## -4.0377 -0.6398 -0.0218  0.5973  5.5820
##
## Random effects:
##  Groups   Name        Variance Std.Dev. Corr
##  ID       (Intercept)  5.583   2.363
##           condition0   6.114   2.473    -0.79
##           condition5   5.980   2.445    -0.61  0.67
##  Residual             18.918   4.350
## Number of obs: 170327, groups:  ID, 33
##
## Fixed effects:
##                     Estimate Std. Error      df t value Pr(>|t|)
## (Intercept)          -3.6323     0.5913 31.0080  -6.143 8.16e-07 ***
## 0TMR                  4.1659     0.6192 31.0054   6.728 1.58e-07 ***
## 5TMR                  4.2235     0.6125 31.0089   6.895 9.87e-08 ***
## non-mus              -1.3041     0.8238 31.0081  -1.583   0.1236
## 0TMR:non-mus          2.2453     0.8627 31.0063   2.603   0.0141 *
## 5TMR:non-mus          1.2101     0.8534 31.0108   1.418   0.1662
## ---
## Signif. codes:  0 '***' 0.001 '**' 0.01 '*' 0.05 '.' 0.1 ' ' 1
```

The model intercept: *Group: musicians, Condition: Speech-in-Quiet*
